# Editorial: Light, clock, flowering, and hormone pathways in attaining abiotic stress tolerance

**DOI:** 10.3389/fpls.2023.1215517

**Published:** 2023-06-22

**Authors:** Madhusmita Panigrahy

**Affiliations:** ^1^ School of Biological Sciences, National Institute of Science Education and Research (NISER), Bhubaneswar, Odisha, India; ^2^ Training School Complex, Homi Bhabha National Institute (HBNI), Mumbai, India

**Keywords:** phytochromes, circadian clock, flowering time, salinity, methylation, jasmonate, coleoptile, photoperiod

Improving yield of food crops is most challenging since yield is the most dynamic trait influenced by various environmental, genetic factors, also due to decrease of yield is pervasive by continuously deteriorating environment in the present-day scenario (Dwivedi et al., 2013). Abiotic and biotic stresses impose great impediments on plant growth and crop productivity (Kumar, 2020). Hence, there has been continuous researches to find various ways not only to combat biotic and abiotic stress, to generate stress resistant plants and crops, but also to attain deeper understanding of different pathways to gain stress resistance and improve crop productivity. Light, and hormone pathways have long been proved to have control over plant yield and stress tolerance (Bechtold and Field, 2018). However, plant circadian clock has also recently been observed to have involvement in these mechanisms (Sharma et al., 2022). Hence, in depth understanding and utilization of recent advances on light, circadian clock and hormone pathways may unlock new roadways to develop strategies for generating abiotic, biotic stress tolerant plants with sustainable or higher yield.

Recent advances such as Phytochrome B discovered as the thermos-sensor in addition to its primary role as red light photoreceptor (Chen et al., 2022), Phytochrome interacting factors (PIF) being the master downstream connectors to thermo-morphogenesis, skoto-morphogenesis, abiotic stress tolerance and flowering pathways (de Lucas and Prat, 2014), discovery of new UV-B photoreceptor UVR8 (Liang et al., 2019), necessity of light for proper root growth (Villacampa et al., 2022), revelation of phytochrome nuclear bodies as active sites of chromatin remodeling and pre-mRNA processing (Cheng et al., 2021), involvement of Phytochrome B in many pathways including abiotic & biotic stresses, herbicide tolerance (Dalazen and Merotto Jr., 2016), flooding tolerance (Courbier and Pierik, 2019), stem mechanical strength (Luo et al., 2022), gravitropism (Xie et al., 2019) are crucial areas to investigate for improving yield. Updates on circadian clock signaling such as evening complex (Ezer et al., 2017), differential regulation of florigen for different photoperiods (Tylewicz et al., 2015), variation of clock organization in different plant systems (Patnaik et al., 2022) are important for designing new stress tolerant strategies in plants. Discovery of noble growth regulators such as strigolactones (Bhatla et al., 2018), phytomelatonin (Moustafa-Farag et al., 2020), and new signaling molecules like nitric oxide (Hancock and Neill, 2019) have opened multiple doors to investigate ways to improve yield of crop plants.

The study by Cortleven et al. shows how alterations in photoperiod induces a stress similar to pathogen stress in plants. They show that photoperiod stress induces transcriptional changes in jasmonic acid and salicylic acid signaling and their synthesis, which are generally observed after pathogen infection. The open question on how pre-treatment on plants having photoperiod stress increasepathogen resistance should be investigated in further experiments. Photoperiod stress enhances pathogen defence response could be extended for deeper understanding following to facts shown by Cortleven et al..

A recent update on results of time lag between temperature and light cycles and their effects on the circadian clock and can be predicted by its entrainment properties is shown by Masuda et al.. The authors use transgenic *Lectuca sativa* seedlings with a luciferase reporter system to demonstrate this with a phase oscillator model in simulation. Based on their predictions, it is now possible to control growth of the plant by adjusting the time lag. Projected leaf area could be used to evaluate the effect of time lag on both growth and circadian rhythm.

As Zhao et al. enclose here an updated research evidence on how light plays a role in maize mesocotyl and coleoptile elongation and germination, as the mesocotyl and coleoptile are considered as two major traits in maize. The authors show that dynamics of different phytohormones accumulation and lignin deposition are closely related during the light-mediated de-etiolation process. Authors also perform transcriptional analysis and establish gene co-expression network, which reveals 49 hub genes in one and 19 hub genes in two modules in this light-mediated process. They lay a robust theoretical foundation of the molecular network underlying the inhibition of maize plasticity elongation by MES and COL in red, blue, and white light stimulations, further functional analysis of promising target and gene will now be easier while extending the research in gene editing and breeding applications.

Patnaik et al. investigate the role of GIGENTEA in response to *Fusarium oxysporum* infection is at molecular level by comparing in different mutant backgrounds of *Arabidopsis thaliana*. The result of this study shows that jasmonic acid pathway is up-regulated post infection during wilt disease caused due to *F.oxysporum*. The confirmatory evidence of Patnaik et al. on involvement of GIGENTEA, component of circadian clock, in biotic stress tolerance has built a strong fundamental base to perform further experiments of control of diseases in crops by controlling clock in crop plants.

Importance of N6-methylation of messenger RNA for the photomorphogenic responses is shown by Zhang et al.. The authors study profiles the transcriptome of William 82 cultivar of soybean in response to light. The authors show that light signaling pathway genes such as GmSPA1, GmPRR5 and GmIC6 undergo methylation in response to light. They also claim that differential m6A peaks are involved in photosynthesis and circadian rhythm pathways. This comprehensive map of light-regulated m6A modification in soybean by Zhang et al. lays a solid foundation for further research into the functional role of light on RNA m6A modification in soybean.

## Author contributions

The author confirms being the sole contributor of this work and approved it for publication.

## References

[B1] Bechtold U. Field B. (2018). Molecular mechanisms controlling plant growth during abiotic stress. J. Exp. Bot. 69 (11), 2753–2758. doi: 10.1093/jxb/ery157 29788471 PMC5961130

[B2] Bhatla S. C. Lal A. M. and Bhatla S. C. (2018). Recently discovered plant growth regulators. Plant Physiol. Dev. Metab., 681–728. doi: 10.1007/978-981-13-2023-1_22

[B3] Chen D. Lyu M. Kou X. Li J. Yang Z. Gao L.. (2022). Integration of light and temperature sensing by liquid-liquid phase separation of phytochrome b. Mol. Cell 82 (16), 3015–3029. doi: 10.1016/j.molcel.2022.05.026 35728588

[B4] Cheng M. C. Kathare P. K. Paik I. Huq E. (2021). Phytochrome signaling networks. Annu. Rev. Plant Biol. 72, 217–244. doi: 10.1146/annurev-arplant-080620-024221 33756095 PMC10988782

[B5] Courbier S. Pierik R. (2019). Canopy light quality modulates stress responses in plants. Iscience 22, 441–452. doi: 10.1016/j.isci.2019.11.035 31816531 PMC6909002

[B6] Dalazen G. Merotto A. Jr. (2016). Physiological and genetic bases of the circadian clock in plants and their relationship with herbicides efficacy. Planta Daninha 34, 191–198. doi: 10.1590/S0100-83582016340100020

[B7] de Lucas M. Prat S. (2014). PIF s get BR right: PHYTOCHROME INTERACTING FACTOR s as integrators of light and hormonal signals. New Phytol. 202 (4), 1126–1141. doi: 10.1111/nph.12725 24571056

[B8] Dwivedi S. Sahrawat K. Upadhyaya H. Ortiz R. (2013). Food, nutrition and agrobiodiversity under global climate change. Adv. Agron. 120, 1–128. doi: 10.1016/B978-0-12-407686-0.00001-4

[B9] Ezer D. Jung J. H. Lan H. Biswas S. Gregoire L. Box M. S.. (2017). The evening complex coordinates environmental and endogenous signals in arabidopsis. Nat. Plants 3 (7), 1–12. doi: 10.1038/nplants.2017.87 PMC5495178 28650433

[B10] Hancock J. T. Neill S. J. (2019). Nitric oxide: its generation and interactions with other reactive signaling compounds. Plants 8 (2), 41. doi: 10.3390/plants8020041 30759823 PMC6409986

[B11] Kumar S. (2020). Abiotic stresses and their effects on plant growth, yield and nutritional quality of agricultural produce. Int. J. Food Sci. Agric. 4, 367–378. doi: 10.26855/ijfsa.2020.12.002

[B12] Liang T. Yang Y. Liu H. (2019). Signal transduction mediated by the plant UV-b photoreceptor UVR8. New Phytol. 221 (3), 1247–1252. doi: 10.1111/nph.15469 30315741

[B13] Luo F. Zhang Q. Xin H. Liu H. Yang H. Doblin M. S.. (2022). A phytochrome b-PIF4-MYC2/MYC4 module inhibits secondary cell wall thickening in response to shaded light. Plant Commun. 3 (6), 100416. doi: 10.1016/j.xplc.2022.100416 35927944 PMC9700123

[B14] Moustafa-Farag M. Elkelish A. Dafea M. Khan M. Arnao M. B. Abdelhamid M. T.. (2020). Role of melatonin in plant tolerance to soil stressors: salinity, pH and heavy metals. Molecules 25 (22), 5359. doi: 10.3390/molecules25225359 33212772 PMC7696660

[B15] Patnaik A. Alavilli H. Rath J. Panigrahi K. C. Panigrahy M. (2022). Variations in circadian clock organization & function: a journey from ancient to recent. Planta 256 (5), 91. doi: 10.1007/s00425-022-04002-1 36173529

[B16] Sharma M. Irfan M. Kumar A. Kumar P. Datta A. (2022). Recent insights into plant circadian clock response against abiotic stress. J. Plant Growth Regul. 41 (8), 3530–3543. doi: 10.1007/s00344-021-10531-y

[B17] Tylewicz S. Tsuji H. Miskolczi P. Petterle A. Azeez A. Jonsson K.. (2015). Dual role of tree florigen activation complex component FD in photoperiodic growth control and adaptive response pathways. Proc. Natl. Acad. Sci. 112 (10), 3140–3145. doi: 10.1073/pnas.1423440112 25713384 PMC4364234

[B18] Villacampa A. Fañanás-Pueyo I. Medina F. J. Ciska M. (2022). Root growth direction in simulated microgravity is modulated by a light avoidance mechanism mediated by flavonols. Physiol. Plantarum 174 (3), e13722. doi: 10.1111/ppl.13722 PMC9327515 35606933

[B19] Xie C. Zhang G. An L. Chen X. Fang R. (2019). Phytochrome-interacting factor-like protein OsPIL15 integrates light and gravitropism to regulate tiller angle in rice. Planta 250, 105–114. doi: 10.1007/s00425-019-03149-8 30927053

